# Effect of equilibration time on clinical and neonatal outcomes in human blastocysts vitrification

**DOI:** 10.1002/rmb2.12328

**Published:** 2020-05-08

**Authors:** Shingo Mitsuhata, Momoko Hayashi, Yoshitaka Fujii, Hiroaki Motoyama, Yuji Endo

**Affiliations:** ^1^ IVF Center Kurashiki Medical Clinic Kurashiki Japan

**Keywords:** blastocyst, clinical and neonatal outcomes, cryoprotectant, equilibration time, vitrification

## Abstract

**Purpose:**

Prolonged exposure to equilibration solutions may be detrimental to an embryo's developmental potential, whereas a shorter exposure may affect the penetration of cryoprotectants into blastomeres. The purpose of this study was to evaluate the effects of different equilibration times on the clinical and neonatal outcomes of human blastocyst vitrification.

**Methods:**

This is a retrospective study based on data collected between November 2008 and November 2015. A total of 192 blastocysts (80 non‐expanded and 112 expanded) obtained from 167 patients were analyzed. The blastocysts were divided into two groups according to their equilibration time: 8‐11 minutes or 12‐15 minutes. The clinical and neonatal outcomes of warmed blastocysts were evaluated.

**Results:**

The survival, implantation, and live birth rates of non‐expanded blastocysts were not different between the two groups, but they significantly improved for the expanded blastocysts in the 12‐15 minutes group compared to the 8‐11 minutes group. The results were similar for the neonatal outcomes after vitrified embryo transfer, when partitioned by equilibration time and blastocyst stage at vitrification.

**Conclusions:**

For the non‐expanded blastocysts, a shortened equilibration time (8‐11 minutes) is sufficient for effective vitrification.

## 1 INTRODUCTION

To date, vitrified blastocyst transfer has been widely conducted. Blastocyst formation is a form of selection for embryos with improved viability,^1^ and vitrified‐warmed cycles may benefit from better endometrial receptivity and enhanced synchronization between embryonic and endometrial development. Accordingly, there is an increasing need to cryopreserve human blastocysts.

A two‐step protocol is generally utilized for human embryo vitrification. First, embryos are exposed to an equilibration solution containing a low concentration of cryoprotectants (CPAs). Second, embryos are exposed to a vitrification solution containing a relatively high concentration of CPAs. The stepwise exposure of embryos to different concentrations of CPAs is thought to be very important for the success of vitrification ^2^; however, the cytotoxicity to the blastomere of the CPAs contained in the equilibration and vitrification solutions is unclear. Commercial vitrification media often contains the CPAs ethylene glycol (EG) and dimethyl sulfoxide (DMSO).

A critical consideration is the duration of blastocyst suspension in the vitrification solution, which should be strictly controlled within 1 minute to reduce the cytotoxic effects of the highly concentrated CPAs. On the other hand, the duration of blastocyst suspension in the equilibration solution is flexible. To simplify the vitrification process, some embryologists adopt a fixed equilibration time (Table 1). These differences suggest that there is no universally agreed upon equilibration duration for human blastocysts, thus leading us to pose the following question: To what extent does the equilibration time influence the clinical outcomes of human blastocyst vitrification? Moreover, knowledge is still limited regarding the safety of vitrification in terms of neonatal outcomes of babies delivered from vitrified embryos.

**Table 1:** Studies showing different equilibration time of blastocyst vitrification and their clinical outcomes

Study	Species	Loading device	Embryo age at vitrification	Equilibration time	Vitrification time	Temperature	Survival rate	Implantation rate	Live birth rate
Tong et al ^3^	Human	Cryotop	D5 or D6 blastocyst	8‐10 min	60 s	RT	‐	47.0%	‐
Cobo et al ^4^	Human	Cryotop	D5 or D6 blastocyst	10‐12 min	45 s	RT	96.6%	42.3%	36.8%
Mori et al ^5^	Human	Cryotop	D5 or D6 blastocyst^a^	15 min	90 s	RT	100.0%	‐	‐
Coello et al ^6^	Human	Cryotop	D5 or D6 blastocyst	12 min	45 s	RT	97.2%	42.0%	‐

Abbreviation: RT, room temperature.

^a^ Blastocysts used in this study were already vitrified and warmed once.

Prolonged exposure to equilibration solutions may be harmful to an embryo's developmental potential, whereas a shorter exposure may affect the penetration of CPAs into blastomeres. The purpose of this study was to evaluate the effects of different equilibration times on the clinical and neonatal outcomes of human blastocyst vitrification.

## 2 MATERIALS AND METHODS

### 2.1 Experimental data

In this retrospective observational study, data were collected between November 2008 and November 2015 from 167 infertility patients. Causes of infertility included male factors, female factors (oviduct and endometrial), and/or unexplained infertility. This study was approved by the Ethics Committee at Kurashiki Medical Center, Japan, and was conducted electronically via a web site, which included additional explanatory information and an opt‐out option.

### 2.2 Blastocyst preparations

In the oocyte retrieval cycle, ovarian stimulation was achieved using standard gonadotropin‐releasing hormone and agonist/follicle‐stimulating hormone (FSH) protocols or an antagonist/FSH protocol. Vaginal ultrasound‐guided follicle puncture was conducted 36 hours after an injection of human chorionic gonadotropin (Mochida, Tokyo, Japan). The retrieved oocytes were inseminated by conventional in vitro fertilization or intracytoplasmic sperm injection in accordance with a previously reported method.^7^, ^8^ The oocytes with two pronuclei and a second polar body were defined as normally fertilized at 17‐19 hours after insemination. Embryos were cultured for 5 days in media (global^®^; Life Global) supplemented with recombinant human albumin (G‐MM; Vitrolife, Gothenburg, Sweden) at 37°C in 6% CO_2_, 5% O_2_, and 89% N_2_. On day 5, the blastocysts were evaluated for expansion. A morphological grade was assigned before blastocyst vitrification according to the Istanbul consensus criteria.^9^ Blastocysts with blastocoels that occupied less than half of the embryo volume and with no signs of zona thinning or expansion were categorized as non‐expanded blastocysts and were <150 µm in diameter. On the other hand, blastocysts with zona thinning and blastocoels that occupied greater than half of the embryo volume were identified as expanded blastocysts. This category also included hatching blastocysts and these embryos were ≧150 µm in diameter. The development of the inner cell mass (ICM) and trophectoderm (TE) was assessed. The ICM grading was as follows: A: tightly packed, many cells; B: loosely grouped, several cells; and C: very few cells. The TE grading was as follows: A: many cells forming a tightly knit epithelium; B: few cells; and C: very few cells forming a loose epithelium. These scores differentiated between “good” (AA), “fair” (AB, BA, BB, AC, CA), and “poor” (BC, CB, CC) graded blastocysts.

### 2.3 Blastocyst vitrification and warming

The Cryotop^®^ method (Kitazato Cryotop^®^ method; Kitazato Corporation, Shizuoka, Japan) was used for blastocyst vitrification and has been described elsewhere.^10^ Blastocysts were loaded into the equilibration solution for 8‐11 minutes or 12‐15 minutes, and sequentially transferred to the vitrification solution for 60 seconds. The blastocysts were immediately placed on the Cryotop^®^ strip with a minimal amount of vitrification solution and were quickly immersed into liquid nitrogen (LN_2_). After several months’ storage in LN_2_, the blastocysts were warmed in thawing solution for 2 minutes and sequentially transferred to dilution solution for 3 minutes. The blastocysts were then washed in a washing solution for 5 minutes. Collapsing procedures of the blastocoel such as artificial shrinkage or trophectoderm biopsy and assisted hatching procedures were not performed on any embryos.

### 2.4 Transfer of post‐warmed blastocysts

Blastocyst survival was defined as a blastocyst that was partially intact after warming and re‐expansion following culture in vitro before transfer. Each surviving blastocyst was transferred to a patient's uterus. Common modalities for blastocyst transfer were natural cycles or hormonal replacement cycles for endometrial preparation. Blastocyst transfer was performed under ultrasound guidance using an embryo transfer (ET) catheter.

### 2.5 Follow‐up and evaluation index

To confirm the establishment of a clinical pregnancy, an ultrasound examination was performed in order to visualize a gestational sac and a fetal heartbeat. The loss of a fetus with a gestational age of <20 weeks was considered a spontaneous abortion. The live birth rate was calculated by dividing the number of live birth delivery cycles by the number of transfer cycles. A preterm birth was defined as <37 gestational weeks. A birth weight <2500 g was defined as low birth weight. The duration of pregnancy, mode of delivery, and weight and sex of the child were recorded as neonatal outcomes.

### 2.6 Statistical analysis

The primary outcome was the live birth rate per warming cycle. Secondary outcomes were embryo survival and implantation rates. Analyses were performed using the Statcel 2 program (OMS Publishing). Continuous variables are represented as means ± SD. When the continuous variables were normally distributed, an F‐test was applied to compare the equality of variances in order to decide whether to use Student's *t* test (which assumes that the two populations are normally distributed with equal variances) or Welch's *t* test (which is applied for unequal variances but maintains the assumption of normality). When the continuous variables were abnormally distributed, the Mann‐Whitney U test was used to compare the 8‐11 and 12‐15 minutes groups. Categorical variables were each described as a frequency and percentage, with between‐group differences tested by the chi‐squared test or Fisher's exact test when the expected frequencies were <5. The 95% confidence interval (CI) was determined using an online calculator. *P* < .05 was considered statistically significant.

## 3 RESULTS

In all cycles, single ET was completed. The average interval time from warming to transfer was comparable for the two groups (8‐11 and 12‐15 minutes groups) of non‐expanded and expanded blastocysts (14.5 ± 6.7 vs 14.5 ± 6.7 hours and 15.1 ± 7.0 vs 13.1 ± 7.5 hours, respectively). The average equilibration duration for the 8‐11 minutes group in non‐expanded and expanded blastocysts was 9.65 ± 0.86 and 9.65 ± 0.86 minutes, and for the 12‐15 minutes group in non‐expanded and expanded blastocysts was 13.7 ± 1.25 and 13.4 ± 1.34 minutes. There were no significant differences among the groups.

Patient characteristics are summarized in Table 2. Characteristics of the two groups (8‐11 and 12‐15 minutes) of non‐expanded and expanded blastocysts were comparable regarding the mean age of patients (35.1 ± 5.1 vs 35.1 ± 4.4 years and 33.0 ± 4.0 vs 32.7 ± 4.0 years), the number of oocyte pickup trial cycles (1.9 ± 1.8 vs 1.9 ± 1.9 and 1.4 ± 0.8 vs 1.3 ± 0.6), and the average number of supernumerary vitrified blastocysts (3.6 ± 1.9 vs 3.8 ± 2.1 and 5.0 ± 3.9 vs 5.0 ± 2.3), respectively. The causes of infertility such as oviduct factor (5.1% vs 3.0% and 11.1% vs 18.0%), endometrial factor (2.6% vs 3.0% and 6.7% vs 10.0%), male factor (20.5% vs 15.2% and 31.1% vs 22.0%), combination factor (10.3% vs 6.1% and 6.7% vs 12.0%), and unexplained factor (61.5% vs 72.7% and 44.4% vs 38.0%), respectively.

**Table 2:** Demographic of patients of non‐expanded and expanded blastocysts following 8‐11 and 12‐15 minutes’ equilibration vitrification protocols

Characteristics	Non‐expanded blastocysts		Expanded blastocysts	
	8‐11 min	12‐15 min	8‐11 min	12‐15 min
Women	39	33	45	50
Age^a^ (mean ± SD)	35.1 ± 5.1	35.1 ± 4.4	33.0 ± 4.0	32.7 ± 4.0
OPU trials (mean ± SD)	1.9 ± 1.8	1.9 ± 1.9	1.4 ± 0.8	1.3 ± 0.6
Cause of infertility				
Oviduct (per women)	2 (5.1%)	1 (3.0%)	5 (11.1%)	9 (18.0%)
Endometrial (per women)	1 (2.6%)	1 (3.0%)	3 (6.7%)	5 (10.0%)
Male (per women)	8 (20.5%)	5 (15.2%)	14 (31.1%)	11 (22.0%)
Combination (per women)	4 (10.3%)	2 (6.1%)	3 (6.7%)	6 (12.0%)
Unexplained (per women)	24 (61.5%)	24 (72.7%)	20 (44.4%)	19 (38.0%)
Vitrified blastocysts	144	151	256	297
Supernumerary vitrified blastocysts (mean ± SD)	3.6 ± 1.9	3.8 ± 2.1	5.0 ± 3.9	5.0 ± 2.3

Note

No significant difference. Values presented as number.

Abbreviation: OPU, oocyte pick up.

^a^ Women age when own embryo was vitrified.

### 3.1 Clinical outcomes of non‐expanded and expanded blastocysts

In all cycles, 192 total blastocysts were warmed, from which 43 babies were born (live birth rate: 22.4%).

Results for the two groups (8‐11 and 12‐15 minutes) of non‐expanded blastocysts were comparable in terms of the endometrial environment during the vitrified blastocyst transfer program, with a natural cycle (77.5% vs 70.0%, 95% CI: 0.55‐3.95) or hormonal replacement cycle (22.5% vs 30.0%, 95% CI: 0.25‐1.82).

The rates of ICM and TE grades—good (32.5% vs 27.5%, 95% CI: 0.49‐3.27), fair (60.0% vs 55.0%, 95% CI: 0.51‐2.96), and poor (7.5% vs 17.5%, 95% CI: 0.10‐1.49)—were also comparable between the two groups. In the 8‐11 minutes group of non‐expanded blastocysts, 40 blastocysts were warmed and 39 (97.5%) survived. In the 12‐15 minutes group, 40 blastocysts were warmed and 38 (95.0%) survived. There were no significant differences in survival rates between the groups (95% CI: 0.26‐16.2). The rates of implantation (20.0% vs 20.0%, 95% CI: 0.34‐2.91) and live birth (12.5% vs 17.5%, 95% CI: 0.20‐2.23) were comparable between the two groups.

Results for the two groups (8‐11 and 12‐15 minutes) of expanded blastocysts were comparable in terms of the endometrial environment during the vitrified blastocyst transfer program, with a natural cycle (76.9% vs 83.3%, 95% CI: 0.27‐1.67) or hormonal replacement cycle (23.1% vs 16.7%, 95% CI: 0.60‐3.76).

The rates of ICM and TE grades—good (69.2% vs 71.7%, 95% CI: 0.40‐2.00), fair (28.8% vs 25.0%, 95% CI: 0.53‐2.78), and poor (1.9% vs 3.3%, 95% CI: 0.07‐4.50)—were also comparable between the two groups. In the 8‐11 minutes group of expanded blastocysts, 52 blastocysts were warmed and 46 (88.5%) survived. In the 12‐15 minutes group, 60 blastocysts were warmed and 59 (98.3%) survived. The survival rate was significantly higher in the 12‐15 minutes group compared to the 8‐11 minutes group (95% CI: 1.16‐50.0, *P* < .05). Additionally, the implantation rate was significantly higher in the 12‐15 minutes group (45.0%) compared to the 8‐11 minutes group (23.1%). The 95% CI was 1.21‐6.14 (*P* < .05). Furthermore, the live birth rate was significantly improved in the 12‐15 minutes group (38.3%) compared to the 8‐11 minutes group (15.4%). The 95% CI was 1.39‐8.31 (*P* < .05). The details are summarized in Table 3.

**Table 3:** Clinical outcomes of non‐expanded and expanded blastocysts following 8‐11 and 12‐15 minutes’ equilibration vitrification protocols

	Non‐expanded blastocysts			Expanded blastocysts		
Warming cycle results	8‐11 min	12‐15 min	95% CI	8‐11 min	12‐15 min	95% CI
Warming cycles	40	40	‐	52	60	‐
Type of endometrial preparation						
Natural cycles (per warming cycles)	31 (77.5%)	28 (70.0%)	0.55‐3.95	40 (76.9%)	50 (83.3%)	0.27‐1.67
HRT cycles (per warming cycles)	9 (22.5%)	12 (30.0%)	0.25‐1.82	12 (23.1%)	10 (16.7%)	0.60‐3.76
Warmed blastocysts	40	40	‐	52	60	‐
Blastocyst morphology^a^						
Good (per warmed blastocysts)	13 (32.5%)	11 (27.5%)	0.49‐3.27	36 (69.2%)	43 (71.7%)	0.40‐2.00
Fair (per warmed blastocysts)	24 (60.0%)	22 (55.0%)	0.51‐2.96	15 (28.8%)	15 (25.0%)	0.53‐2.78
Poor (per warmed blastocysts)	3 (7.5%)	7 (17.5%)	0.10‐1.49	1 (1.9%)	2 (3.3%)	0.07‐4.50
Survived blastocysts (per warmed blastocysts)	39 (97.5%)	38 (95.0%)	0.26‐16.2	46 (88.5%)*	59 (98.3%)*	1.16‐50.0
Implantation^b^ (per warmed blastocysts)	8 (20.0%)	8 (20.0%)	0.34‐2.91	12 (23.1%)**	27 (45.0%)**	1.21‐6.14
Live birth (per warmed blastocysts)	5 (12.5%)	7 (17.5%)	0.20‐2.23	8 (15.4%)***	23 (38.3%)***	1.39‐8.31

Note

Values presented as number.

Abbreviations: CI, confidence interval; HRT, hormone replacement therapy.

^a^ Blastocyst morphology when the blastocyst was vitrified.

^b^ Defined as a gestational sac identified with ultrasound.

* *P* < .05; **P < .05; ***P < .05.

### 3.2 Neonatal outcomes of non‐expanded and expanded blastocysts

The neonatal outcomes of 43 children born after blastocyst transfer are summarized in Table 4.

**Table 4:** Neonatal outcomes of non‐expanded and expanded blastocysts following 8‐11 and 12‐15 minutes’ equilibration vitrification protocols

Delivered baby data	Non‐expanded blastocysts			Expanded blastocysts		
	8‐11 min	12‐15 min	95% CI	8‐11 min	12‐15 min	95% CI
Babies	5	7	‐	8	23	‐
Gestational length (weeks; mean ± SD)	38.2 ± 0.8	38.6 ± 1.1	‐1.7‐1.0	37.9 ± 1.2	38.4 ± 1.7	‐1.8‐0.9
Premature birth (per babies)	0 (0%)	0 (0%)	‐	0 (0%)	2 (8.7%)	0‐5.84
Birth weight (g; mean ± SD)	3068.0 ± 154.3	3342.0 ± 326.3	‐627.5‐79.5	3017.3 ± 429.2	3082.0 ± 339.3	‐
Low birth weight (per babies)	0 (0%)	0 (0%)	‐	0 (0%)	0 (0%)	‐
Cesarean section (per babies)	1 (20.0%)	3 (42.9%)	0.03‐3.80	3 (33.3%)	11 (47.8%)	0.14‐3.17
Proportion of male babies (per babies)	3 (60.0%)	4 (57.1%)	0.12‐9.82	4 (50.0%)	12 (52.2%)	0.20‐4.26
Congenital abnormalities (per babies)	0 (0%)	0 (0%)	‐	0 (0%)	0 (0%)	‐

Note

No significant difference. Values presented as number.

Abbreviation: CI, confidence interval.

For the non‐expanded blastocysts, 5 babies were born in the 8‐11 minutes group and 7 babies were born in the 12‐15 minutes group. There were no differences between the 8‐11 minutes and 12‐15 minutes groups with respect to the average gestational length (38.2 ± 0.8 vs. 38.6 ± 1.1 weeks, 95% CI: −1.7‐1.0), preterm birth rate (0% vs. 0%), mean birth weight (3068.0 ± 154.3 vs. 3342.0 ± 326.3 g, 95% CI: −627.5‐79.5), and low birth weight rate (0% vs. 0%). In addition, the rates of cesarean section (20.0% vs. 42.9%, 95% CI: 0.03‐3.80), the proportion of male babies (60.0% vs. 57.1%, 95% CI: 0.12‐9.82), and incidence of congenital abnormalities (0% vs. 0%) were also not different.

For the expanded blastocysts, 8 babies were born in the 8‐11 minutes group and 23 were born in the 12‐15 minutes group. There were no differences between the 8‐11 minutes and 12‐15 minutes groups with respect to the average gestational length (37.9 ± 1.2 vs. 38.4 ± 1.7 weeks, 95% CI: −1.8‐0.9), the preterm birth rate (0% vs. 8.7%, 95% CI: 0‐5.84), the mean birth weight (3017.3 ± 429.2 vs. 3082.0 ± 339.3 g), and the low birth weight rate (0% vs. 0%). Moreover, the rates of cesarean section (33.3% vs. 47.8%, 95% CI: 0.14‐3.17), proportion of male babies (50.0% vs. 52.2%, 95% CI: 0.20‐4.26), and incidence of congenital abnormalities (0% vs. 0%) were also not different.

## 4 DISCUSSION

Both equilibration times (8‐11 minutes and 12‐15 minutes) in the non‐expanded blastocyst group resulted in very effective vitrification. However, in the expanded blastocyst group, the shorter equilibration time (8‐11 minutes) worsened clinical outcomes, whereas an increase in the equilibration time to 12‐15 minutes improved clinical outcomes. There was no effect on overall neonatal outcomes.

A two‐step protocol is generally utilized to vitrify human embryos. The commercial media's protocols often recommend that the equilibration time depends on the recovery of the shape of the embryo, which can be assessed by visualization of the shrinkage and swelling of the blastomere under a stereomicroscope. However, when vitrifying embryos at the blastocyst stage, it is often difficult to assess whether shrinkage or expansion has occurred, and some blastocysts will not shrink in the vitrifying media. In our laboratory, four embryos were vitrified for 15 minutes. The exposure was dependent on the embryo and the embryologist's skills; the final embryo's vitrification was completed within 15 minutes. All equilibration times were recorded and we determined what the exposure time for individual blastocysts was according to the record. Kitazato's protocol recommends a maximum embryo exposure time to the equilibration solution of 12 minutes at the cleavage stage and 15 minutes at the morulae and blastocyst stage. Our findings are in agreement that a 12‐ minutes threshold is one of the criteria in evaluating the permeability of the equilibration solution, and the vitrified blastocysts should be divided into two groups based on an equilibration time over or under 12 minutes.

In the non‐expanded blastocysts, both datasets conclusively demonstrated that effective vitrification had occurred. According to Vanderzwalmen et al,^11^ since non‐expanded blastocysts contain blastocoels of small volumes, the concentration of CPAs usually increases faster inside the cells, and the initial amount of liquid is reduced. This allows for sufficient permeation of the CPAs and a more rapid equilibration before vitrification. Non‐expanded blastocysts in this study were identified when the blastocoels occupied less than half of the embryo volume; they were < 150 µm in diameter. Consequently, the non‐expanded blastocysts survived after the vitrification procedure. Unnecessary prolonged CPA exposure could have harmful effects to embryonic development, so a shorter exposure to equilibration solution is preferable for non‐expanded blastocyst vitrification.

In the expanded blastocysts, a shorter equilibration time (8‐11 minutes) during vitrification was detrimental to clinical outcomes, whereas results improved with an increased equilibration time of 12‐15 minutes. As blastocysts develop, both their blastocoel volume and cell number increase, increasing the need for maximum equilibration with CPAs. A short exposure time to the CPAs results in a low concentration of permeable CPAs present inside the cavity, which is likely insufficient to protect the blastocysts against the formation of ice crystals inside the blastocoel. Vanderzwalmen et al ^11^ suggested that an insufficient permeation of CPAs inside the cavity might cause ice crystal formation during the cooling step, thereby reducing post‐warming survival. In this study, expanded blastocysts were identified when the blastocoels occupied greater than half of the embryo volume; they were ≧ 150 µm in diameter. Collapsing procedures of blastocoels such as artificial shrinkage or trophectoderm biopsy were not performed on any embryos; expanded blastocysts were vitrified with the large‐volume blastocoel cavity. In the 8‐11 minutes group of expanded blastocysts, the survival rate was 86.7%. Mukaida et al ^12^ studied the vitrification of day 5 blastocysts using the Cryoloop and reported an 87% survival rate. Rama et al ^13^ suggested vitrifying day 5 blastocysts using Cryoloop and reported a survival rate of 80.6%. In these studies, blastocyst equilibration time to low concentration CPAs was 2 minutes. Furthermore, Darwish et al ^14^ reported vitrifying day 5 blastocysts using Cryoleaf with a survival rate of 74.9%. In this study, the blastocyst equilibration time to low concentration CPAs ranged from 6 to 10 minutes. With shorter equilibration times, the survival rate decreased. This study also suggested that the blastocoel might be insufficiently dehydrated after a shorter equilibration time of 8‐11 minutes such that much longer exposures to equilibration solution are needed.

Kader et al ^15^ assessed the effect of equilibration time on the DNA integrity index (DII) of vitrified‐warmed mouse blastocysts. The DII of the non‐expanded blastocysts significantly improved with 8‐ and 15‐ minutes equilibration protocols compared to a 4‐ minutes protocol. Moreover, the DII of the expanded blastocysts significantly improved with an 8‐ minutes protocol compared to 4‐ or 15‐ minutes protocols. However, these results must be interpreted with caution because mouse blastocysts are smaller than human embryos and thus may react differently to vitrification. Nonetheless, this animal study suggested that equilibration time is an important parameter in embryo cryopreservation.

Studies have recorded no differences in gestational age, birth weight, proportion of male babies, rates of cesarean section, or congenital abnormalities. Takahashi et al ^16^ demonstrated that the use of CPAs, (such as 15% EG in an equimolar mixture with DMSO) had no negative effects on the perinatal outcomes of blastocyst transfer using vitrification when compared to fresh blastocyst transfer. Rama et al ^13^ reported that there were no statistically significant differences in the mean gestational length, incidence of preterm deliveries, mean birth weight, mean Apgar score, or the incidence of congenital birth defects between vitrified day 3 embryos and fresh ETs. However, their sample size was too small to provide sufficient statistical validity. Shi et al ^17^ analyzed the outcomes of 494 babies delivered after vitrified ET and showed that all perinatal and obstetric parameters were similar between vitrified and fresh embryos. In these studies, within each type of vitrification method, variations can be found in media composition, equilibration and dilution time, carrier devices, embryo stage, and warming methods. Our findings are important in confirming the safety of vitrification for clinical use and serve to counter recent published concerns about the use of CPAs for vitrification and their purported negative effects on organogenesis.

We acknowledge that the manipulation skills of each embryologist may affect the study's outcomes. However, in this investigation, only embryologists with more than 5 years’ clinical experience undertook embryo vitrification, and their results were statistically comparable. Therefore, the differences in technique between embryologists probably did not influence the study's outcomes.

In conclusion, a short equilibration time (8‐11 minutes) is sufficient for blastocyst vitrification when the blastocoel cavity is small. However, these results should be interpreted with caution due to the small study size and the high risk of bias. Further randomized controlled trials that examine clinical and neonatal outcomes are necessary to adequately judge the efficacy and safety of vitrification.

## CONFLICT OF INTEREST

Shingo Mitsuhata, Yoshitaka Fujii, Yuji Endo, Momoko Hayashi and Hiroaki Motoyama declare that they have no conflict of interest.

## HUMAN RIGHTS STATEMENT AND INFORMED CONSENT

All the procedures were followed in accordance with the ethical standards of the institutional ethical committee and with the Helsinki Declaration of 1964 and its later amendments. All the study's participants provided informed consent, and the study design was approved by the appropriate ethics committee of Kurashiki Medical Clinic, Okayama, Japan.

## ANIMAL STUDIES

This article does not contain any studies with animal participants performed by any of the authors.
